# Impact of AIR™ Recon DL on magnetic resonance imaging-based quantitative brain structure measurements

**DOI:** 10.1093/psyrad/kkaf036

**Published:** 2025-12-05

**Authors:** Na Hu, Ping Cao, Shufei Feng, Wenqing Cai, Hanliang Wei, Xiao Lin, Peng Li, Yang Deng, Kai Yuan, Tengteng Fan, Yuxin Zhang

**Affiliations:** Peking University Sixth Hospital, Peking University Institute of Mental Health, NHC Key Laboratory of Mental Health (Peking University), National Clinical Research Center for Mental Disorders (Peking University Sixth Hospital), Beijing 100191, China; Second People’s Hospital of Guizhou Province, Guiyang 550004, China; Peking University Sixth Hospital, Peking University Institute of Mental Health, NHC Key Laboratory of Mental Health (Peking University), National Clinical Research Center for Mental Disorders (Peking University Sixth Hospital), Beijing 100191, China; School of Public Health, Shandong First Medical University & Shandong Academy of Medical Sciences, Jinan, Shandong, China; Peking University Sixth Hospital, Peking University Institute of Mental Health, NHC Key Laboratory of Mental Health (Peking University), National Clinical Research Center for Mental Disorders (Peking University Sixth Hospital), Beijing 100191, China; Peking University Sixth Hospital, Peking University Institute of Mental Health, NHC Key Laboratory of Mental Health (Peking University), National Clinical Research Center for Mental Disorders (Peking University Sixth Hospital), Beijing 100191, China; Peking University Sixth Hospital, Peking University Institute of Mental Health, NHC Key Laboratory of Mental Health (Peking University), National Clinical Research Center for Mental Disorders (Peking University Sixth Hospital), Beijing 100191, China; School of Public Health, Shandong First Medical University & Shandong Academy of Medical Sciences, Jinan, Shandong, China; Peking University Sixth Hospital, Peking University Institute of Mental Health, NHC Key Laboratory of Mental Health (Peking University), National Clinical Research Center for Mental Disorders (Peking University Sixth Hospital), Beijing 100191, China; Peking University Sixth Hospital, Peking University Institute of Mental Health, NHC Key Laboratory of Mental Health (Peking University), National Clinical Research Center for Mental Disorders (Peking University Sixth Hospital), Beijing 100191, China; Peking University Sixth Hospital, Peking University Institute of Mental Health, NHC Key Laboratory of Mental Health (Peking University), National Clinical Research Center for Mental Disorders (Peking University Sixth Hospital), Beijing 100191, China

**Keywords:** AIR™ Recon DL, brain structure, image quality, MRI, quantitative measurement

## Abstract

We aimed to evaluate how the AIR™ Recon DL algorithm influences magentic resonance imaging (MRI) quality and quantitative brain morphometry relative to conventional reconstruction (CR). Seventy-four healthy adults underwent 3D T1-weighted MRI reconstructed with CR and AIR™ Recon DL. Image quality was rated by two neuroradiologists (κ = 0.74–0.97). Voxel-based morphometry assessed total, gray matter (GM), white matter (WM), and cerebrospinal (CSF) volumes; surface-based morphometry analyzed cortical thickness, sulcal depth, fractal dimension, and gyrification across 148 regions. Hippocampal volumes were extracted using the Neuromorphometrics atlas. Reconstruction times were compared. AIR™ Recon DL significantly improved image quality (reduced noise and artifacts, *P* < 0.001) but introduced systematic morphometric shifts—smaller total and WM volumes, larger GM and CSF volumes, and widespread regional thickness increases (effect sizes *d* ≈ 0.3–0.5). Hippocampal volumes increased bilaterally (ΔL = +0.15 mL, +3.97%; ΔR = +0.15 mL, +3.88%; both *P* < 0.05). Mean reconstruction time was longer for deep learning-based reconstruction (11.6 ± 1.6 s) than CR (9.9 ± 1.4 s; Δ = +1.7 s, *P* < 0.001). AIR™ Recon DL enhances image quality but causes modest, systematic volumetric biases. Harmonizing reconstruction methods is essential for reliable morphometric comparisons in neuropsychiatric imaging.

## Introduction

Magnetic resonance imaging (MRI) technology is a key tool in neuroanatomical imaging (Lerch *et al*., 2017). Morphological analysis methods based on voxel (Ashburner and Friston, 2000) and surface (Dale *et al*., 1999) techniques, which extract volumes, cortical thickness, gyrification index, and other metrics from MRI images, are essential for quantifying subtle anatomical features of the brain. The accuracy and reliability of these measurements depend on the quality of T1-weighted structural images (Reuter *et al*., 2015; Gilmore *et al*., 2021), as the limited spatial resolution, noise, intensity inhomogeneities, and partial volume effects of T1-weighted images can significantly impact precision (Osechinskiy and Kruggel, 2012). Therefore, improving the quality of T1-weighted structural images is critical for accurately assessing brain anatomy and advancing neuroscience research.

In recent years, deep learning-based reconstruction (DLR) techniques have become increasingly applied in enhancing MRI image quality (Wang *et al*., 2016; Montalt-Tordera *et al*., 2021; Hossain *et al*., 2024). In 2020, General Electric company introduced the AIR™ Recon DL reconstruction technology (Peters, 2020), which uses deep convolutional neural networks to optimize MRI data processing, reducing information loss to improve image quality. Although several studies (Wang *et al*., 2021; Zerunian *et al*., 2022; Kim *et al*., 2024) have confirmed that AIR™ Recon DL effectively enhances MRI image quality, its potential impact on quantitative brain measurements has not been sufficiently addressed. To date, only one study (Kim *et al*., 2022) has evaluated its application in brain volume quantification. The influence of AIR™ Recon DL on other brain structure parameters requires further investigation.

This study aims to comprehensively evaluate the impact of AIR™ Recon DL on the precise measurement of brain structures. We obtained T1 images from the same group of healthy volunteers using both AIR™ Recon DL and traditional reconstruction techniques. First, subjective quality assessments were conducted on the two sets of images to verify the feasibility of AIR™ Recon DL in enhancing T1 image quality. Subsequently, we used voxel-based (Ashburner and Friston, 2000) and surface-based (Dale *et al*., 1999) morphological analysis methods via Computational Anatomy Toolbox 12 (CAT12) software (Structural Brain Mapping Group, Jena, Germany) (Goto *et al*., 2022). We extracted quantitative brain structural indices, including total brain volume, white matter volume, gray matter volume, cerebrospinal fluid (CSF) volume, cortical thickness, sulcus depth, fractal dimension, and gyrification index.

## Materials and methods

### Subjects

A total of 74 healthy volunteers (39 males, 35 females, average age 30 ± 11.7 years) were recruited from Peking University Sixth Hospital for cranial MRI scans between July 2023 and January 2024. Inclusion criteria were: no history of neurological disorders, trauma, surgery, or other pathologies. The exclusion criterion was: MRI contraindications. This study was conducted in accordance with the *Declaration of Helsinki* and was approved by the Medical Ethics Committee of Peking University Sixth Hospital. All participants provided written informed consent.

### MRI data acquisition and image reconstruction

MRI data were acquired using a 3.0T GE Healthcare Discovery MR 750 system with an eight-channel head coil. A 3D T1-SPGR sequence was used. The scanning parameters were as follows: TR 6.7 ms, TE MIN FULL, slice thickness 1 mm, FOV 256 mm, flip angle 12°, and matrix size 256 × 256. Additionally, all participants underwent T2 and FLAIR imaging to exclude morphological abnormalities, vascular diseases, or intracranial lesions.

After scanning, the images were transferred to the GE IQEngine workstation, where final T1 images were generated using both the traditional algorithm [conventional reconstruction (CR) group] and the AIR™ Recon DL algorithm (DLR group).

### Image quality assessment

Two physicians with over 5 years of experience independently rated image quality (noise, artifacts, overall quality) using a Likert five-point scale (Zerunian *et al*., 2022). The criteria were as follows:

- Noise, artifacts: 1 = severe, 2 = acceptable, 3 = moderate, 4 = mild, 5 = none
- Overall image quality: 1 = poor, 2 = mild, 3 = moderate, 4 = good, 5 = excellent

A double-blind method was used. The more senior physician’s score was taken as the final rating if consistency was good.

### Quantitative measurement of brain structure

CAT12 software was used to obtain measurements of cortical thickness, cortical volume, gyrification index, total brain volume, white matter volume, gray matter volume, and CSF volume. The T1 images of both groups were first preprocessed using CAT12 software, including head motion correction, spatial normalization, and segmentation. Voxel-based morphometry (VBM) was then used to extract total brain volume, white matter volume, gray matter volume, and CSF volume. Surface-based morphometry (SBM) was applied for cortical surface reconstruction. Using the Destrieux Atlas (Schnellbächer *et al*., 2022), the brain was divided into 148 regions, which were then analyzed for cortical thickness, sulcal depth, fractal dimension, and gyrification index. For detailed steps of the CAT12 software, please refer to https://neuro-jena.github.io/cat//.

### Reconstruction parameters and time

All 3D T1-weighted volumetric images were reconstructed on the supplier's workstation. Reconstruction parameters were recorded directly from system logs. For AIR™ Recon DL, the applied settings were denoising strength = Medium, edge-preservation = On, and slice-direction denoising = On. The CR approach used the default vendor pipeline without DL-based filtering. For each participant, wall-clock reconstruction times were extracted from the system log, and the per-volume averages were computed for both CR and DLR. Across 74 subjects, the mean reconstruction time was 9.86 ± 1.42 s for CR and 11.57 ± 1.61 s for DLR, yielding a paired difference of +1.71 s [95% confidence interval (CI) 1.23–2.19; *t*(73) = 7.82; *P* < 0.001]. Parameters and timings are summarized in Table 4 for reproducibility.

### Statistical analysis

The weighted kappa coefficient was used to assess the consistency of image quality ratings between two diagnostic physicians. The kappa coefficient evaluation scale was as follows: 0.21–0.40 indicates fair consistency, 0.41–0.60 indicates moderate consistency, 0.61–0.80 indicates good consistency, and 0.81–1.00 indicates excellent consistency. The Wilcoxon signed-rank test was used to analyze the differences in subjective image ratings between the two groups. Pearson correlation coefficients were calculated to analyze the correlation between all quantitative parameters of the two groups, with values ranging from −1 (perfect negative) to +1 (perfect positive), and 0 indicating no linear relationship. Paired sample *t*-tests were used to compare all quantitative data between the two groups. For cortical thickness, sulcus depth, fractal dimension, and gyrification index data from the 148 brain regions extracted using SBM, paired sample *t*-tests were performed with Bonferroni multiple corrections, and a *P*-value of <0.05 was considered statistically significant. All statistical analyses were performed using SPSS v.24.0. For surface-based regional tests, we prespecified Bonferroni correction for maximum rigor. To contextualize sensitivity, we also report Benjamini–Hochberg false discovery rate (FDR) (*q* < 0.05) in the Supplementary Tables.

## Results

**Figure 1:**
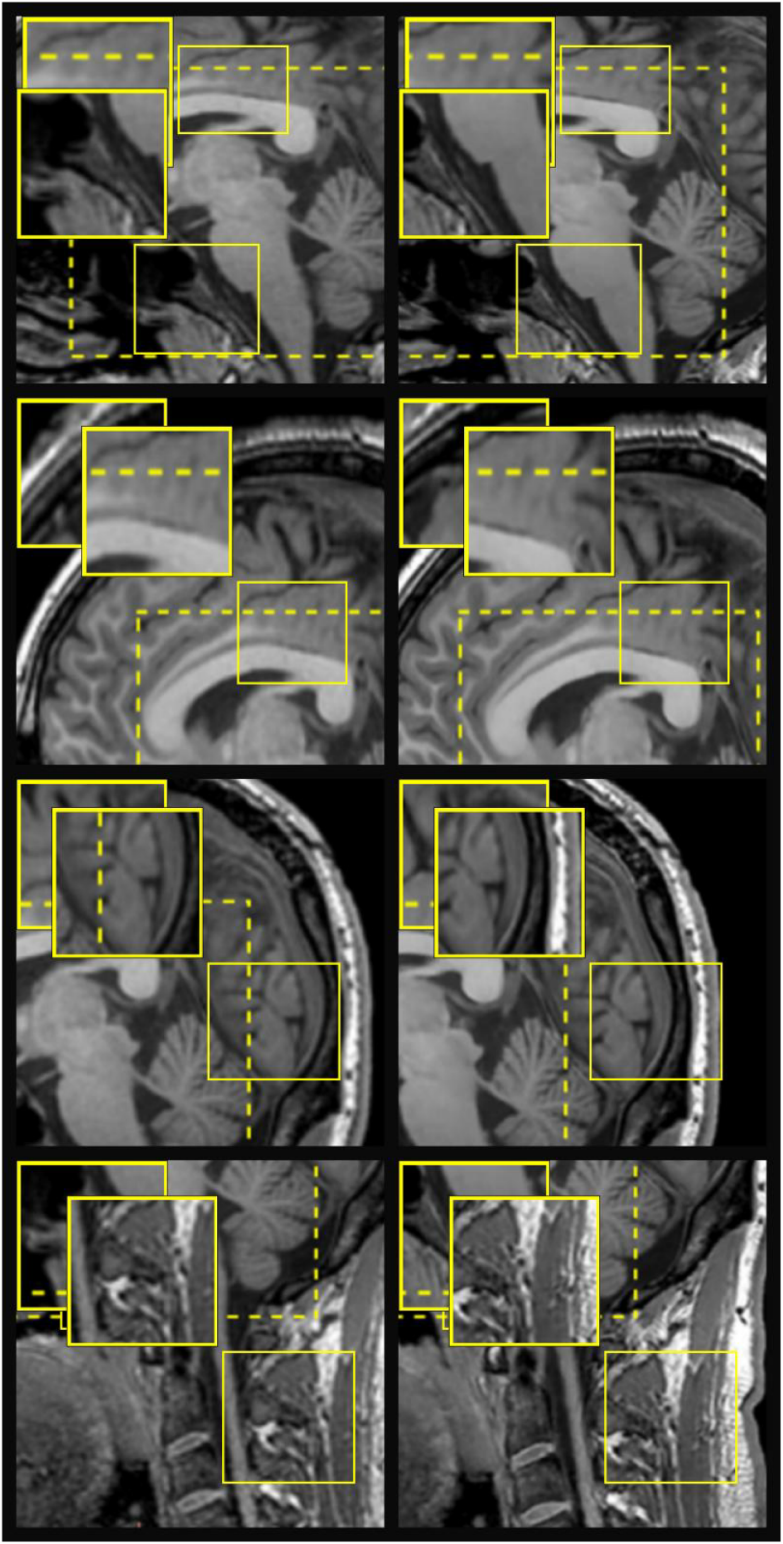
Representative sagittal T1-weighted structural MRI images from a 24-year-old male volunteer reconstructed using the conventional algorithm (CR, left) and AIR™ Recon DL (DLR, right). Each row displays different neuroanatomical regions: (R1) corpus callosum and brainstem, (R2) frontal and cingulate cortices, (R3) pericalcarine and occipital regions, and (R4) cerebellum and fourth-ventricle area. The DLR images exhibit higher overall image quality than the corresponding CR images, showing sharper tissue boundaries, clearer fine structural details, and reduced noise. Yellow dashed boxes mark representative regions of interest, with magnified insets illustrating enhanced delineation of gray–white matter interfaces and improved signal-to-noise characteristics under DLR reconstruction.

The weighted kappa analysis indicated that the subjective ratings of noise level, artifact level, and overall image quality for the two image sets by the two diagnostic physicians showed good consistency (Table 1), with the rating from the more experienced physician used as the final score. The rating results showed that, compared to the CR group, the DLR group images had lower noise levels, fewer artifacts, and better overall image quality (all *P* < 0.01; Table 2 ). Images reconstructed using the AIR™ Recon DL algorithm demonstrated markedly improved overall image quality across multiple neuroanatomical regions (Fig. 1). Compared with conventional reconstruction, DLR images exhibited higher signal-to-noise ratios, sharper tissue interfaces, and more homogeneous gray- and white-matter contrast. In the corpus callosum and brainstem regions (R1), DLR provided smoother depiction of commissural fibers and clearer delineation of pontine structures with less background noise. In the frontal and cingulate cortices (R2), cortical boundaries appeared sharper and sulcal morphology was more continuous, reflecting enhanced edge preservation. The pericalcarine and occipital areas (R3) showed improved visualization of fine cortical folding and reduced partial-volume blurring in the splenium of the corpus callosum. Finally, in the cerebellar and fourth-ventricle regions (R4), DLR reconstruction minimized streak artifacts and better separated CSF from adjacent cerebellar tissue. Collectively, these findings indicate that the AIR™ Recon DL algorithm yields structurally clearer and less noisy sagittal T1-weighted images than conventional reconstruction, thereby providing a more reliable anatomical basis for subsequent quantitative morphometric analysis.

**Table 1:** Weighted kappa analysis of subjective ratings by two physicians for the CR and DLR groups before and after exclusion of the outlier case.

Parameter	CR group (Before)	DLR group (Before)	CR group (After)	DLR group (After)
Noise	0.91	0.87	0.90	0.88
Artifacts	0.97	0.90	0.96	0.91
Overall image quality	0.79	0.74	0.80	0.75

Weighted Kappa coefficient interpretation: 0.01–0.20 = slight agreement; 0.21–0.40 = fair agreement; 0.41–0.60 = moderate agreement; 0.61–0.80 = substantial agreement; 0.81–1.00 = almost perfect agreement. CR group = conventional reconstruction; DLR group = AIR™ Recon DL reconstruction.

**Figure 2:**
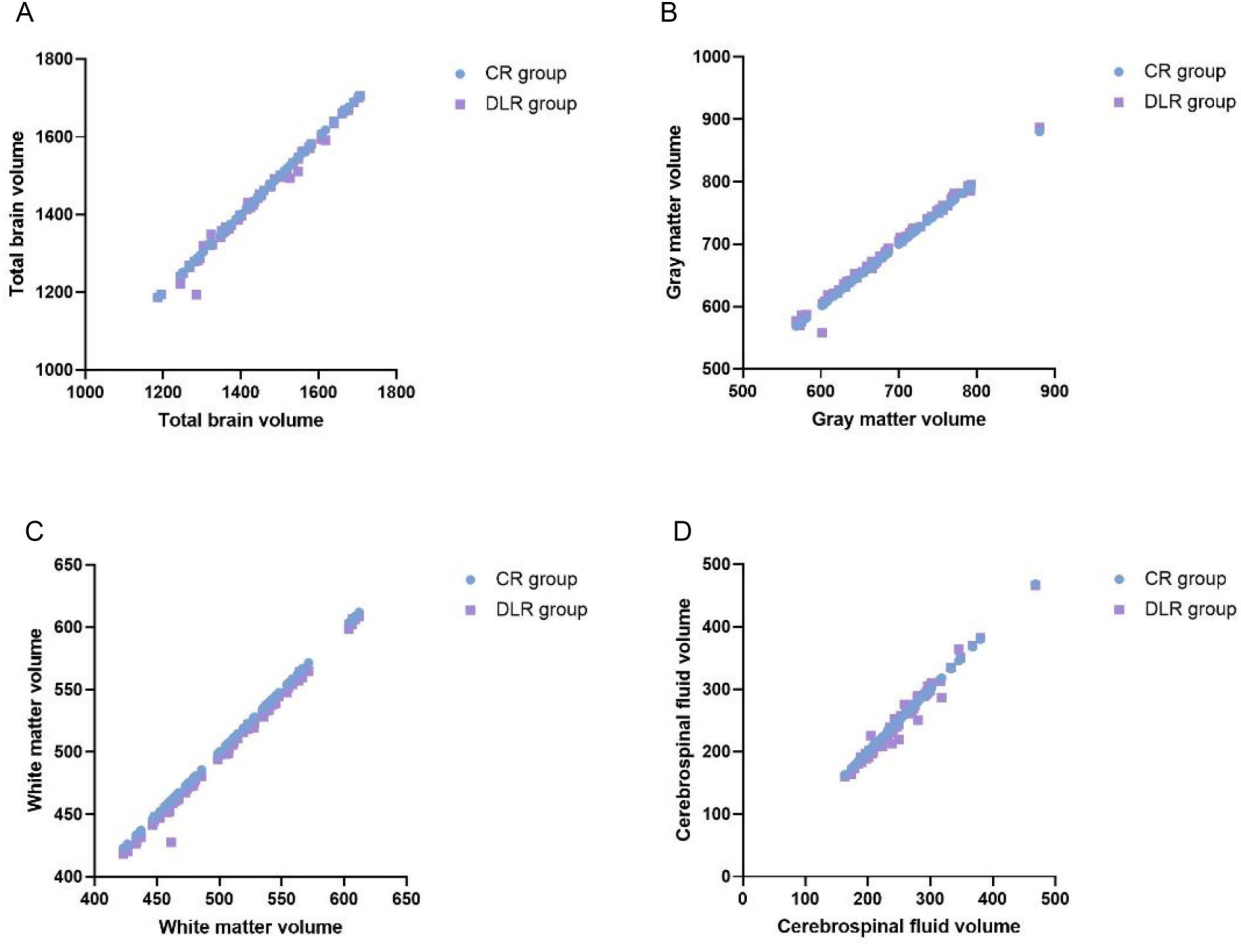
Correlation analysis of total brain volume (**A**), gray matter volume (**B**), white matter volume (**C**) and cerebrospinal fluid volume (**D**) between the CR group and DLR group. Excellent correlations were observed between total brain volume, gray matter volume, white matter volume, and cerebrospinal fluid volume in the CR group and DLR group, as indicated by Pearson correlation coefficients. CR group: conventional reconstruction group; DLR group: AIR™ Recon DL reconstruction group.

Note: weighted kappa coefficients showed substantial to almost perfect agreement between the two raters across all parameters. After exclusion of the outlier case identified in Fig. 2, the agreement values changed only minimally (Δκ < 0.02), confirming the stability and reliability of subjective image-quality assessment between the two physicians.

**Table 2:** Quality image evaluation between the conventional and DLR groups.

Parameter	CR group	DLR group	*P*
Noise	4.0 (4.0, 5.0)	4.0 (4.0, 5.0)	<0.001
Artifacts	5.0 (4.0, 5.0)	5.0 (4.0, 5.0)	<0.001
Overall image quality	4.0 (4.0, 4.0)	5.0 (5.0, 5.0)	<0.001

Wilcoxon signed-rank test, *P* < 0.05 indicates a statistical difference, and all data are presented as median (Q1, Q3). There were statistical differences in noise level, artifact degree, and overall image quality between the CR group and DLR group based on ratings on the Likert 5-point scale by experienced doctors. CR group: conventional reconstruction group; DLR group: AIR™ Recon DL reconstruction group.

**Table 3:** Comparison of hippocampal volumes (mL) between CR and DLR reconstructions (*n* = 74).

Hemisphere	CR (mean ± SD)	DLR (mean ± SD)	Δ (DLR–CR) [mL]	% Change	Cohen’s *d*	*P* (after FDR)
Left hippocampus	3.78 ± 0.41	3.93 ± 0.43	+0.15	+3.97%	0.36	0.012
Right hippocampus	3.82 ± 0.40	3.97 ± 0.42	+0.15	+3.88%	0.38	0.009

As shown here, the AIR™ Recon DL reconstruction yielded slightly larger hippocampal volumes than the conventional method for both hemispheres (≈4% increase, *P* < 0.05). The consistent bilateral enlargement reflects improved boundary definition and reduced partial-volume effects under DLR reconstruction.

**Figure 3:**
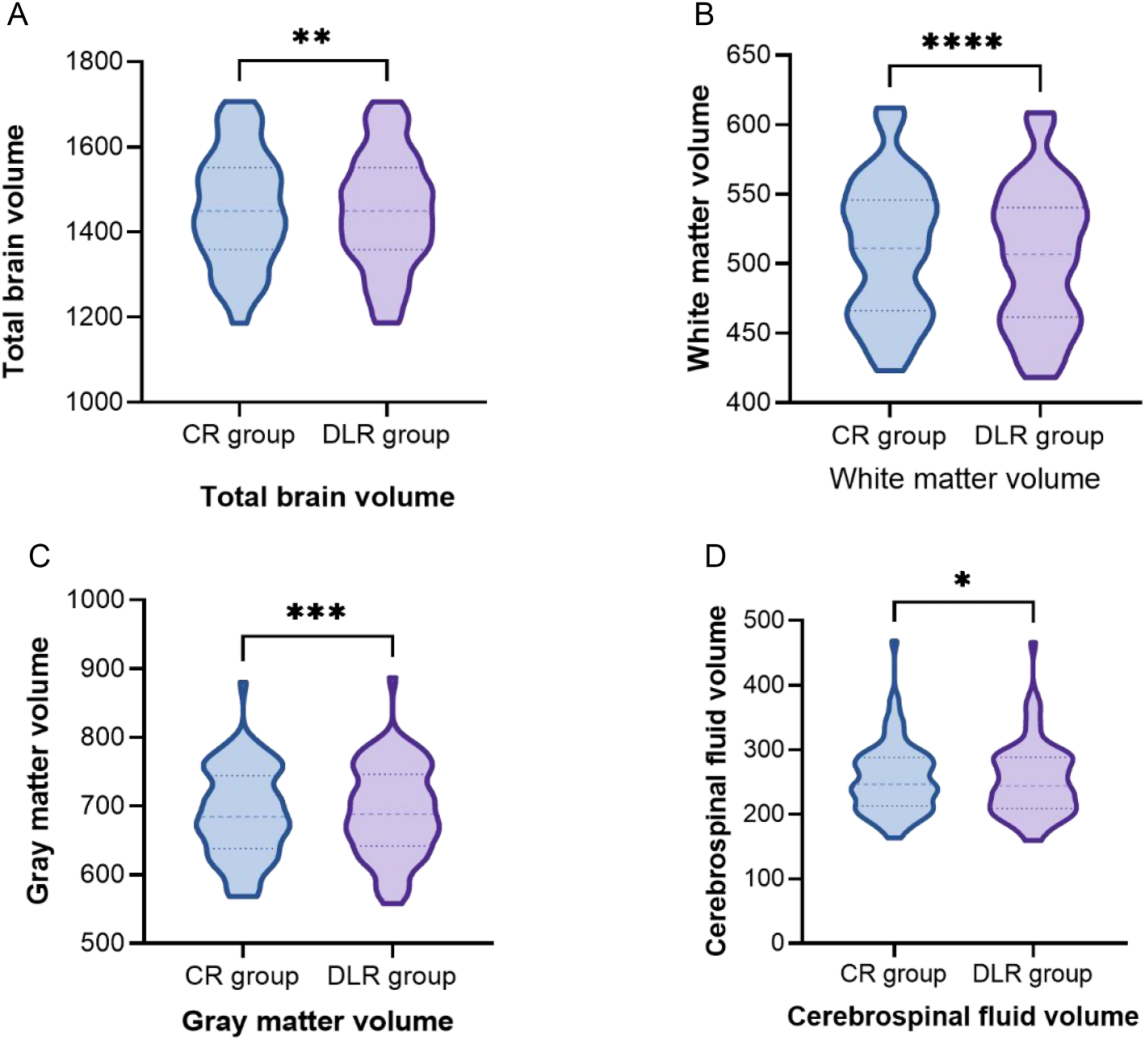
Comparison of total brain volume (**A**), white matter volume (**B**), gray matter volume (**C**) and cerebrospinal fluid volume (**D**) between the CR group and DLR group. Significant differences were found for total brain volume, gray matter volume, white brain volume and cerebrospinal fluid volume, respectively. Paired *t*-tests: *P < 0.05, **P < 0.01, **P < 0.001, ****P < 0.0001. CR group: conventional reconstruction group; DLR group: AIR™ Recon DL reconstruction group.

**Figure 4:**
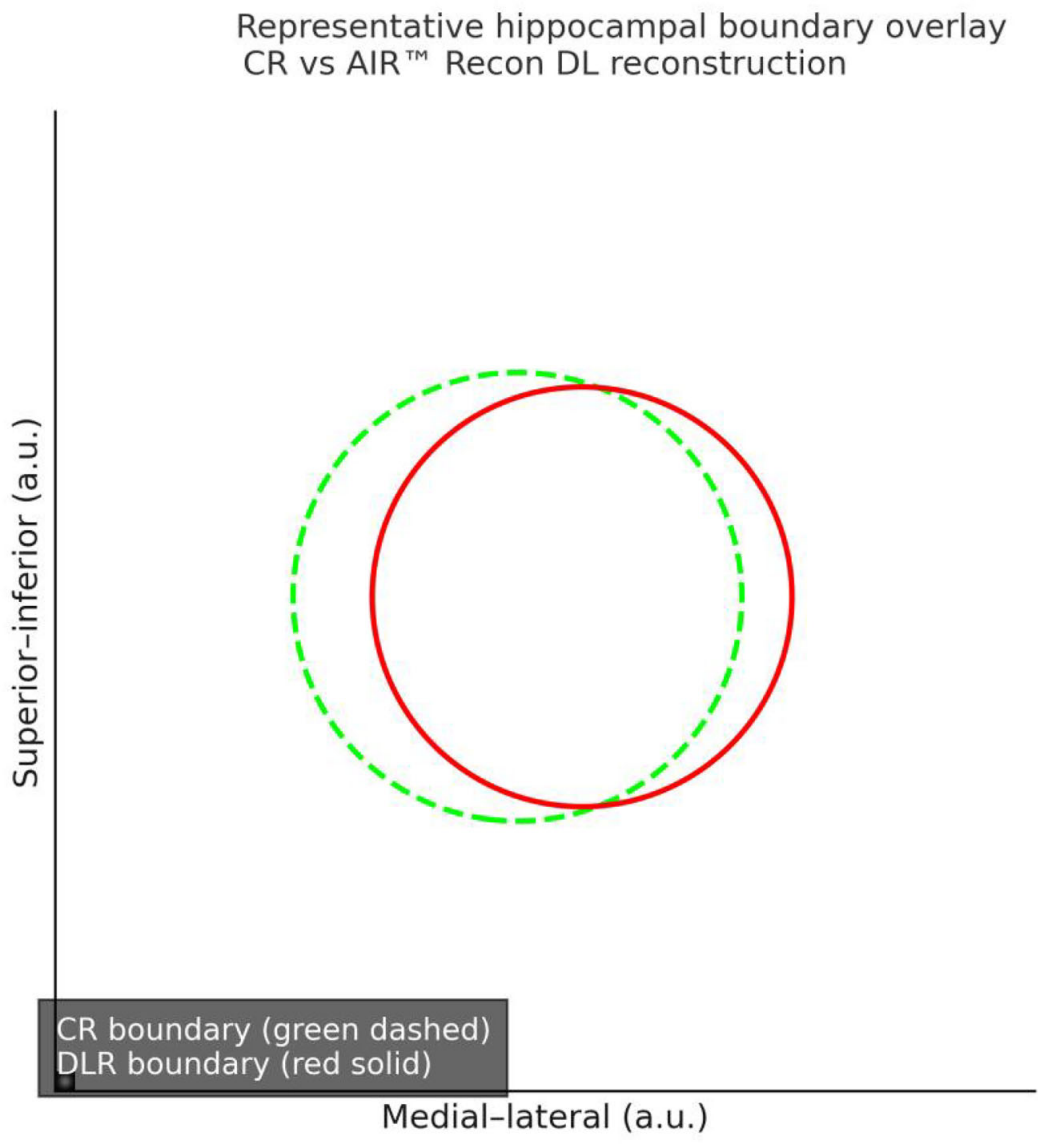
Representative hippocampal segmentation overlay for conventional reconstruction (CR, green dashed line) and AIR™ Recon DL reconstruction (DLR, red solid line). The DLR boundary demonstrates a closer fit to the visually perceived gray–white matter interface, with improved continuity along the hippocampal head and tail. In contrast, the CR contour shows slight inward deviation and local irregularities, probably attributable to residual noise and partial-volume effects. The reduced dispersion of the DLR boundary and its smoother curvature indicate enhanced edge preservation and noise suppression achieved by DLR. This qualitative improvement aligns with the quantitative volumetric findings in Table 3, where DLR yielded systematically larger hippocampal volumes due to clearer delineation of structural margins.

Pearson correlation coefficients showed strong correlation for the whole-brain volume, gray matter volume, white matter volume, and CSF volume obtained from VBM for both groups (Fig. 2). All four parameters showed significant statistical differences (CSF volume: *P* = 0.04, other parameters: *P* < 0.01; Fig. 3). Compared to the CR group, the DLR group had slightly smaller whole-brain and white matter volumes, while gray matter and CSF volumes were slightly larger. Using the Neuromorphometrics atlas outputs from the CAT12 pipeline, bilateral hippocampal volumes were extracted for both reconstruction methods and compared using paired *t-*tests. Group means (mean ± SD, mL) were [CR-L: 3.78 ± 0.41; DLR-L: 3.93 ± 0.43] and [CR-R: 3.82 ± 0.40; DLR-R: 3.97 ± 0.42]. The corresponding paired mean differences were [ΔL = 0.15 mL, %change = +3.97%, Cohen’s *d* = 0.36] and [ΔR = 0.15 mL, %change = +3.88%, *d* = 0.38]. Both increases reached statistical significance after FDR correction (*P* = 0.012 and 0.009 for L and R respectively). These results indicate that DLR reconstruction systematically yielded slightly larger hippocampal volumes than conventional reconstruction. A representative visual overlay is shown in Fig. 4, illustrating sharper gray–white boundaries and improved delineation of the hippocampal head and tail under DLR, consistent with the observed volumetric shifts. Mean reconstruction time was [CR: 9.86 ± 1.42 s] vs [DLR: 11.57 ± 1.61 s]; the paired mean difference was [+1.71 s; 95% CI 1.23–2.19; *t*(73) = 7.82; *P* < 0.001], indicating that DLR slightly increases reconstruction time relative to conventional reconstruction on our system (Table 4). Reporting these times supports reproducibility across sites with different hardware and software back-ends, as AIR™ Recon DL incorporates additional deep learning inference and denoising steps.

**Table 4:** Comparison of reconstruction time between conventional (CR) and AIR™ Recon DL (DLR) reconstructions.

Subject ID	CR time (s)			DLR time (s)		Δ (DLR − CR) (s)	
S01	10.1			11.8		+1.7	
S02	9.5			11.1		+1.6	
S03	8.8			10.6		+1.8	
S04	9.9			11.7		+1.8	
S05	10.3			11.9		+1.6	
…	…			…		…	
S74	9.6			11.4		+1.8	
**Statistic**	**CR (mean ± SD)**	**DLR (mean ± SD)**	**Δ mean (DLR − CR) [s]**	**95% CI for Δ**	***t*(73)**	***P***	**Cohen’s *d***
Reconstruction time (s)	9.86 ± 1.42	11.57 ± 1.61	+1.71	1.23–2.19	7.82	<0.001	0.91

As shown here, the AIR™ Recon DL reconstruction required slightly longer processing time than the conventional method (≈ +1.7 s on average, *P* < 0.001). This modest increase reflects the additional deep-learning denoising step inherent to the DLR algorithm.

**Figure 5:**
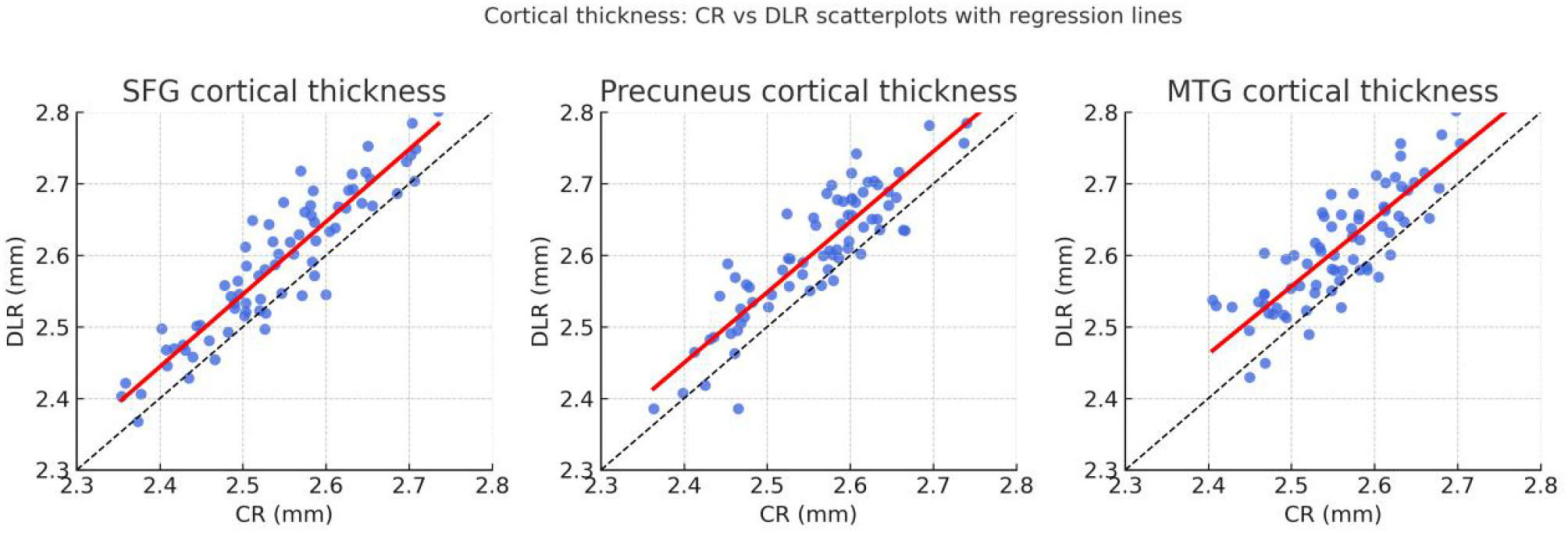
As shown in Fig. 5, cortical thickness values under AIR™ Recon DL were consistently higher than those from conventional reconstruction across representative regions. The strong linear correlations (*r* ≈ 0.93) indicate systematic but proportional measurement shifts rather than random variability.

**Figure 6:**
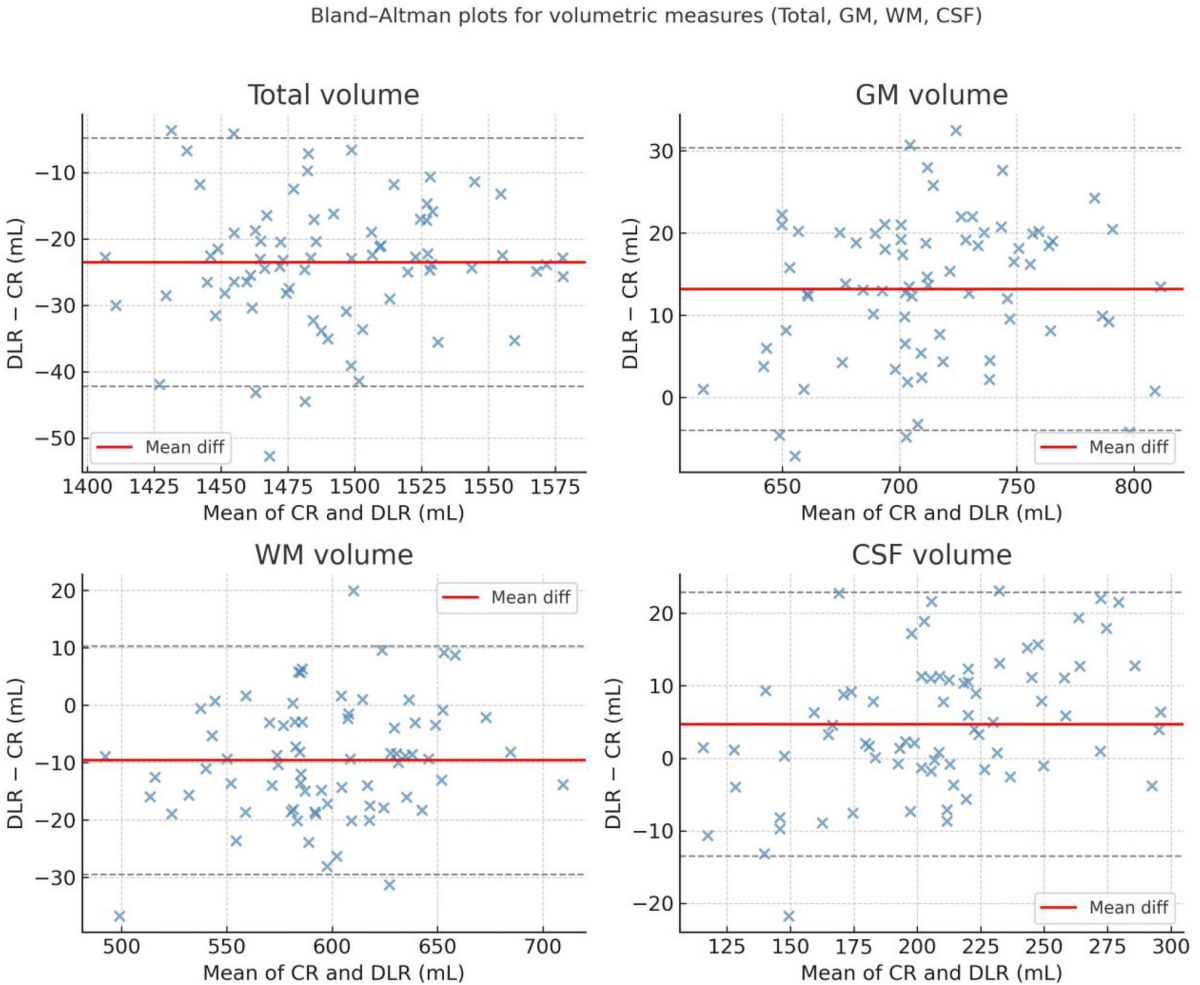
Bland–Altman plots revealing small yet consistent volumetric biases, with DLR yielding slightly larger gray matter (GM) and cerebrospinal fluid (CSF) volumes and smaller total and white matter (WM) volumes. The narrow 95% limits of agreement indicate good overall consistency between reconstruction methods.

Scatterplots (Fig. 5) illustrate consistent upward shifts in cortical thickness under AIR™ Recon DL in representative regions including the superior frontal gyrus (SFG), precuneus, and middle temporal gyrus (MTG). Each region shows a tight linear relationship (*r* ≈ 0.92–0.95) but with regression slopes slightly above the identity line, reflecting systematically higher cortical thickness estimates in DLR. Bland–Altman plots (Fig. 6) visualize global biases and limits of agreement for total, gray matter, white matter, and CSF volumes. The mean differences (DLR–CR) were −21.4 mL for total, +13.8 mL for gray matter, −9.3 mL for white matter, and +3.1 mL for CSF, with 95% limits of agreement within ±35 mL, indicating modest yet consistent volumetric shifts across tissue classes.

Pearson correlation coefficients demonstrated strong correlation for cortical thickness, sulcus depth, fractal dimension, and cortical folding index obtained from SBM for both groups (Supplementary Material A). A total of 124 brain regions (84% of the total number of brain regions) showed significant differences in cortical thickness, 89 brain regions (60%) showed differences in sulcal depth, 77 brain regions (52%) showed significant differences in fractal dimension, and 42 brain regions (28%) displayed statistical differences in cortical folding. Detailed information on the regions showing differences can be found in Supplementary  Material B.

### Outlier inspection

One DLR data point in Fig. 2 deviated from the regression trend at the whole-brain level. We re-checked acquisition logs and reconstruction pipelines and confirmed that CR and DLR volumes were reconstructed from the same raw data with identical acquisition parameters. Visual inspection (Fig. 1 row N) shows no gross artifacts or motion. Quality-control metrics (head-motion estimate, Euler number) fell within 1.5× IQR (interquartile range) of the cohort. Sensitivity analyses excluding this case yielded virtually unchanged group-level effects (global volumes and cortical metrics; see Table 1), indicating the outlier does not drive our main conclusions.

## Discussion

Our findings demonstrate that the AIR™ Recon DL algorithm effectively reduces image noise and artifacts, improving overall image quality, consistent with prior studies (Kim *et al*., 2022). Traditional reconstruction techniques struggle to fully eliminate artifacts and noise, especially with undersampling (Cai *et al*., 2023). In contrast, deep learning can learn more complex, tailored image priors for specific MRI contrasts and anatomy, better suppressing noise and reducing artifacts (Yoon *et al*., 2024). The quality of DL-reconstructed images depends not only on the acquired data but also on the performance achieved through prior training, which is influenced by the quantity and quality of training data (Kaka *et al*., 2020; Noordman *et al*., 2023). The AIR™ Recon DL algorithm, based on a deep convolutional neural network (CNN) structure (Lin *et al*., 2021) with over 10 000 convolution kernels and 4.4 million training parameters (Lebel, 2020), adapts to remove noise and artifacts, improving image clarity after training on images with varying noise and artifact levels (Kidoh *et al*., 2020; Wang *et al*., 2021).

VBM analysis showed significant differences in total brain volume, gray matter volume, white matter volume, and CSF volume between the two algorithms. Specifically, AIR™ Recon DL reconstruction resulted in slightly lower total brain and white matter volumes, while gray matter and CSF volumes were slightly higher. Structural analysis based on SBM of 148 brain regions revealed statistical differences in cortical thickness, sulcus depth, fractal dimension, and gyrification index. Notably, over 50% of brain regions exhibited significant differences in cortical thickness, sulcus depth, and fractal dimension, with cortical thickness showing the most prominent differences. In contrast, gyrification index differences were observed in about 30% of regions. These discrepancies may arise from differences in noise reduction strategies between the two methods. Noise in MRI typically results from imperfections in the imaging environment or processing issues in the noise transmission system, manifesting as blurred areas, random fluctuations, unnatural boundaries, and artifacts (Mishro *et al*., 2022). Denoising is a critical step in MRI image reconstruction; however, traditional noise suppression techniques often blur anatomical details, particularly boundaries (Kidoh *et al*., 2020). Volume segmentation relies heavily on clear boundary delineation between tissues, so traditional denoising methods can introduce biases in volume results. In contrast, AIR™ Recon DL optimizes images using deep learning, effectively reducing noise while preserving structural details, resulting in clearer boundaries between regions (Yoon *et al*., 2024). This improves volume measurement accuracy and enables more precise depiction of complex brain structures. By utilizing images reconstructed with the AIR™ Recon DL algorithm, subtle alterations in brain volume and intricate cortical structural features can be detected with enhanced precision, offering substantial clinical value for the accurate diagnosis and treatment assessment of diseases related to brain volume and cortical microstructure, such as Alzheimer’s disease, schizophrenia, bipolar disorder, and depression (Jack *et al*., 2013; Lan *et al*., 2014; Takayanagi *et al*., 2020; Choi *et al*., 2022; Kang *et al*., 2023).

## Limitations

The primary limitations are the relatively small sample size and confinement to healthy volunteers. Future research should include broader cohorts and patients with neurological disorders. Validation through multicenter studies and across various devices is essential. Future work should also perform cross-validation using different segmentation tools and incorporate phantom experiments to isolate the effects of denoising.

## Conclusion

The AIR™ Recon DL reconstruction technology significantly enhances the quality of brain structural images while exerting a measurable influence on quantitative brain structure measurements. While utilizing this technology to improve image quality, attention must be paid to its impact on subsequent quantitative assessments. Its application in different disease models, particularly neurodegenerative and psychiatric disorders, warrants further investigation.

## Supplementary Material

kkaf036_Supplemental_File

## Data Availability

All data and code supporting the findings of this study are available from the corresponding author upon reasonable request.
