# Supplementary material for: Impact of AIR™ Recon DL on magnetic resonance imaging-based quantitative brain structure measurements
Source: Psychoradiology. 2025 Dec 5;6:kkaf036. doi: 10.1093/psyrad/kkaf036 (PMC12936584; doi:10.1093/psyrad/kkaf036)
Supplement: kkaf036_Supplemental_File [file kkaf036_supplemental_file.docx]

**Impact of AIR™ recon DL on MRI-based quantitative brain structure measurements**

**Table S1. Pearson correlation coefficients for cortical thickness, fractal dimension, sulcus depth, and gyrification index across brain regions**

| Brain Region | Pearson correlation coefficient (r) | | | |
| --- | --- | --- | --- | --- |
|  | cortical thickness | fractal dimension | sulcus depth | gyrification index |
| Left Gyrus and Sulcus, Frontal Margin | 0.9743 | 0.9826 | 0.9865 | 0.6089 |
| Right Gyrus and Sulcus, Frontal Margin | 0.9725 | 0.9739 | 0.9736 | 0.6646 |
| Left Gyrus and Sulcus, Occipital Inferior | 0.9496 | 0.9831 | 0.9758 | 0.6722 |
| Right Gyrus and Sulcus, Occipital Inferior | 0.9670 | 0.9871 | 0.9937 | 0.7314 |
| Left Gyrus and Sulcus, Paracentral | 0.9648 | 0.9894 | 0.9697 | 0.8511 |
| Right Gyrus and Sulcus, Paracentral | 0.9569 | 0.9829 | 0.9508 | 0.8219 |
| Left Gyrus and Sulcus, Subcentral | 0.9814 | 0.9746 | 0.9450 | 0.7523 |
| Right Gyrus and Sulcus, Subcentral | 0.9779 | 0.9926 | 0.9701 | 0.8181 |
| Left Gyrus and Sulcus, Transversal Frontal Pole | 0.9382 | 0.9919 | 0.9880 | 0.7397 |
| Right Gyrus and Sulcus, Transversal Frontal Pole | 0.9666 | 0.9808 | 0.9723 | 0.7020 |
| Left Gyrus and Sulcus, Anterior Cingulate | 0.9847 | 0.9909 | 0.9897 | 0.8778 |
| Right Gyrus and Sulcus, Anterior Cingulate | 0.9762 | 0.9669 | 0.9472 | 0.7945 |
| Left Gyrus and Sulcus, Mid Anterior Cingulate | 0.9662 | 0.9898 | 0.9759 | 0.8153 |
| Right Gyrus and Sulcus, Mid Anterior Cingulate | 0.9674 | 0.9832 | 0.9578 | 0.8092 |
| Left Gyrus and Sulcus, Mid Posterior Cingulate | 0.9528 | 0.9943 | 0.9936 | 0.7525 |
| Right Gyrus and Sulcus, Mid Posterior Cingulate | 0.9642 | 0.9670 | 0.9754 | 0.7493 |
| Left Cingulate, Posterior Dorsal | 0.8908 | 0.9949 | 0.9833 | 0.7786 |
| Right Cingulate, Posterior Dorsal | 0.9164 | 0.9382 | 0.9465 | 0.8106 |
| Left Cingulate, Posterior Ventral | 0.9034 | 0.9889 | 0.9807 | 0.7640 |
| Right Cingulate, Posterior Ventral | 0.8911 | 0.9296 | 0.9720 | 0.7457 |
| Left Gyrus, Cuneus | 0.9255 | 0.9935 | 0.9970 | 0.8191 |
| Right Gyrus, Cuneus | 0.8845 | 0.9770 | 0.9461 | 0.7801 |
| Left Gyrus, Inferior Frontal Opercular | 0.9847 | 0.9508 | 0.9442 | 0.8159 |
| Right Gyrus, Inferior Frontal Opercular | 0.9817 | 0.9907 | 0.9944 | 0.8811 |
| Left Gyrus, Inferior Frontal Orbital | 0.9050 | 0.9185 | 0.9101 | 0.5340 |
| Right Gyrus, Inferior Frontal Orbital | 0.9477 | 0.9837 | 0.9946 | 0.7663 |
| Left Gyrus, Inferior Frontal Triangular | 0.9644 | 0.9803 | 0.9790 | 0.8513 |
| Right Gyrus, Inferior Frontal Triangular | 0.9869 | 0.9913 | 0.9859 | 0.7761 |
| Left Gyrus, Middle Frontal | 0.9854 | 0.9843 | 0.9958 | 0.6927 |
| Right Gyrus, Middle Frontal | 0.9861 | 0.9915 | 0.9877 | 0.7827 |
| Left Gyrus, Superior Frontal | 0.9654 | 0.9862 | 0.9817 | 0.9386 |
| Right Gyrus, Superior Frontal | 0.9638 | 0.9907 | 0.9865 | 0.8198 |
| Left Insula, Large and Central Insular Sulcus | 0.7361 | 0.8702 | 0.9361 | 0.6874 |
| Right Insula, Large and Central Insular Sulcus | 0.8487 | 0.9352 | 0.9901 | 0.5365 |
| Left Insula, Short Gyrus | 0.7436 | 0.9065 | 0.9199 | 0.6496 |
| Right Insula, Short Gyrus | 0.8753 | 0.9231 | 0.9939 | 0.7481 |
| Left Gyrus, Occipital Middle | 0.9875 | 0.9753 | 0.9841 | 0.7577 |
| Right Gyrus, Occipital Middle | 0.9814 | 0.9861 | 0.9868 | 0.8702 |
| Left Gyrus, Occipital Superior | 0.9762 | 0.9928 | 0.9928 | 0.8159 |
| Right Gyrus, Occipital Superior | 0.9621 | 0.9767 | 0.9897 | 0.7640 |
| Left Occipital-Temporal, Lateral Fusiform | 0.9312 | 0.9812 | 0.9708 | 0.7260 |
| Right Occipital-Temporal, Lateral Fusiform | 0.9200 | 0.9679 | 0.9513 | 0.7033 |
| Left Occipital-Temporal, Medial Lingual | 0.8798 | 0.9875 | 0.9890 | 0.8830 |
| Right Occipital-Temporal, Medial Lingual | 0.9179 | 0.9668 | 0.9774 | 0.7217 |
| Left Occipital-Temporal, Medial Parahippocampal | 0.7837 | 0.8229 | 0.8850 | 0.7031 |
| Right Occipital-Temporal, Medial Parahippocampal | 0.6611 | 0.9017 | 0.9477 | 0.5026 |
| Left Gyrus, Orbital | 0.8841 | 0.8515 | 0.9709 | 0.7571 |
| Right Gyrus, Orbital | 0.8977 | 0.9358 | 0.9744 | 0.7646 |
| Left Parietal Inferior, Angular | 0.9798 | 0.9811 | 0.9903 | 0.8795 |
| Right Parietal Inferior, Angular | 0.9807 | 0.9945 | 0.9849 | 0.8146 |
| Left Parietal Inferior, Supramarginal | 0.9885 | 0.9806 | 0.9590 | 0.7966 |
| Right Parietal Inferior, Supramarginal | 0.9773 | 0.9937 | 0.9890 | 0.7679 |
| Left Parietal Superior | 0.9807 | 0.9941 | 0.9907 | 0.8685 |
| Right Parietal Superior | 0.9742 | 0.9912 | 0.9815 | 0.9168 |
| Left Gyrus, Postcentral | 0.9698 | 0.9896 | 0.9711 | 0.7111 |
| Right Gyrus, Postcentral | 0.9755 | 0.9892 | 0.9607 | 0.8166 |
| Left Gyrus, Precentral | 0.9680 | 0.9868 | 0.9858 | 0.7687 |
| Right Gyrus, Precentral | 0.9645 | 0.9890 | 0.9801 | 0.8553 |
| Left Gyrus, Precuneus | 0.9709 | 0.9939 | 0.9937 | 0.8913 |
| Right Gyrus, Precuneus | 0.9661 | 0.9865 | 0.9777 | 0.8698 |
| Left Gyrus, Rectus | 0.9163 | 0.9781 | 0.9935 | 0.7925 |
| Right Gyrus, Rectus | 0.8519 | 0.9529 | 0.8839 | 0.7866 |
| Left Gyrus, Subcallosal | 0.8840 | 0.9038 | 0.9858 | 0.5410 |
| Right Gyrus, Subcallosal | 0.7271 | 0.9269 | 0.9327 | 0.6228 |
| Left Temporal Superior, G_T Transversal | 0.9779 | 0.8458 | 0.9119 | 0.7038 |
| Right Temporal Superior, G_T Transversal | 0.9715 | 0.9666 | 0.9832 | 0.8245 |
| Left Temporal Superior, Lateral | 0.9767 | 0.8766 | 0.9352 | 0.8094 |
| Right Temporal Superior, Lateral | 0.9819 | 0.9843 | 0.9885 | 0.8964 |
| Left Temporal Superior, Planar Polar | 0.7548 | 0.7470 | 0.9490 | 0.5540 |
| Right Temporal Superior, Planar Polar | 0.8574 | 0.9219 | 0.9429 | 0.7450 |
| Left Temporal Superior, Planar Temporal | 0.9823 | 0.9419 | 0.9471 | 0.8652 |
| Right Temporal Superior, Planar Temporal | 0.9867 | 0.9939 | 0.9653 | 0.7842 |
| Left Gyrus, Temporal Inferior | 0.9304 | 0.9328 | 0.9616 | 0.6413 |
| Right Gyrus, Temporal Inferior | 0.9421 | 0.9832 | 0.9282 | 0.6027 |
| Left Gyrus, Temporal Middle | 0.9788 | 0.9563 | 0.9786 | 0.6546 |
| Right Gyrus, Temporal Middle | 0.9791 | 0.9936 | 0.9746 | 0.7196 |
| Left Lateral Fissure, Anterior Horizontal | 0.9622 | 0.9280 | 0.8807 | 0.6708 |
| Right Lateral Fissure, Anterior Horizontal | 0.9773 | 0.9859 | 0.9823 | 0.8047 |
| Left Lateral Fissure, Anterior Vertical | 0.9733 | 0.9668 | 0.9603 | 0.7111 |
| Right Lateral Fissure, Anterior Vertical | 0.9555 | 0.9858 | 0.9856 | 0.8214 |
| Left Lateral Fissure, Posterior | 0.9749 | 0.8874 | 0.8546 | 0.8047 |
| Right Lateral Fissure, Posterior | 0.9382 | 0.9778 | 0.9824 | 0.8055 |
| Left Occipital Pole | 0.9379 | 0.9910 | 0.9909 | 0.8885 |
| Right Occipital Pole | 0.9155 | 0.9727 | 0.9574 | 0.6010 |
| Left Temporal Pole | 0.9156 | 0.7944 | 0.9264 | 0.5410 |
| Right Temporal Pole | 0.8845 | 0.9363 | 0.8738 | 0.7205 |
| Left Sulcus, Calcarine | 0.8771 | 0.9924 | 0.9875 | 0.9028 |
| Right Sulcus, Calcarine | 0.8917 | 0.9401 | 0.9410 | 0.7178 |
| Left Sulcus, Central | 0.9447 | 0.9875 | 0.9780 | 0.8273 |
| Right Sulcus, Central | 0.9438 | 0.9825 | 0.9713 | 0.8850 |
| Left Sulcus, Cingulate Marginalis | 0.9734 | 0.9927 | 0.9957 | 0.8629 |
| Right Sulcus, Cingulate Marginalis | 0.9585 | 0.9748 | 0.9783 | 0.8483 |
| Left Sulcus, Circular Insula Anterior | 0.7637 | 0.9440 | 0.9375 | 0.6393 |
| Right Sulcus, Circular Insula Anterior | 0.9169 | 0.9121 | 0.9881 | 0.7110 |
| Left Sulcus, Circular Insula Inferior | 0.8528 | 0.8819 | 0.9322 | 0.7992 |
| Right Sulcus, Circular Insula Inferior | 0.9244 | 0.9802 | 0.9857 | 0.8549 |
| Left Sulcus, Circular Insula Superior | 0.9592 | 0.9579 | 0.9023 | 0.7766 |
| Right Sulcus, Circular Insula Superior | 0.9615 | 0.9812 | 0.9873 | 0.9056 |
| Left Sulcus, Collateral Transversal Anterior | 0.7507 | 0.8862 | 0.8682 | 0.6443 |
| Right Sulcus, Collateral Transversal Anterior | 0.8838 | 0.8859 | 0.9027 | 0.7720 |
| Left Sulcus, Collateral Transversal Posterior | 0.9288 | 0.9664 | 0.9782 | 0.7307 |
| Right Sulcus, Collateral Transversal Posterior | 0.9475 | 0.9826 | 0.9756 | 0.7323 |
| Left Sulcus, Inferior Frontal | 0.9807 | 0.9886 | 0.9835 | 0.6183 |
| Right Sulcus, Inferior Frontal | 0.9761 | 0.9912 | 0.9944 | 0.8968 |
| Left Sulcus, Middle Frontal | 0.9750 | 0.9918 | 0.9876 | 0.7458 |
| Right Sulcus, Middle Frontal | 0.9787 | 0.9944 | 0.9924 | 0.7044 |
| Left Sulcus, Superior Frontal | 0.9785 | 0.9919 | 0.9947 | 0.8070 |
| Right Sulcus, Superior Frontal | 0.9779 | 0.9922 | 0.9946 | 0.7273 |
| Left Sulcus, Intermittent Primary Jensen | 0.9621 | 0.9701 | 0.9857 | 0.6998 |
| Right Sulcus, Intermittent Primary Jensen | 0.9602 | 0.9906 | 0.9955 | 0.8388 |
| Left Sulcus, Intraparietal and Parietal Transversal | 0.9806 | 0.9824 | 0.9962 | 0.9398 |
| Right Sulcus, Intraparietal and Parietal Transversal | 0.9820 | 0.9850 | 0.9964 | 0.9308 |
| Left Sulcus, Occipital Middle and Lunatus | 0.9674 | 0.9620 | 0.9922 | 0.7371 |
| Right Sulcus, Occipital Middle and Lunatus | 0.9674 | 0.9874 | 0.9945 | 0.8597 |
| Left Sulcus, Occipital Superior and Transversal | 0.9677 | 0.9876 | 0.9922 | 0.8145 |
| Right Sulcus, Occipital Superior and Transversal | 0.9841 | 0.9889 | 0.9893 | 0.8382 |
| Left Sulcus, Occipital Anterior | 0.9738 | 0.9567 | 0.9599 | 0.5848 |
| Right Sulcus, Occipital Anterior | 0.9653 | 0.9853 | 0.9958 | 0.7804 |
| Left Sulcus, Occipital-Temporal Lateral | 0.9564 | 0.9882 | 0.9740 | 0.7812 |
| Right Sulcus, Occipital-Temporal Lateral | 0.9365 | 0.9824 | 0.9834 | 0.6780 |
| Left Sulcus, Occipital-Temporal Medial and Lingual | 0.9310 | 0.9844 | 0.9712 | 0.6821 |
| Right Sulcus, Occipital-Temporal Medial and Lingual | 0.9477 | 0.9415 | 0.9662 | 0.6087 |
| Left Sulcus, Orbital Lateral | 0.9544 | 0.9805 | 0.9796 | 0.5911 |
| Right Sulcus, Orbital Lateral | 0.9645 | 0.9883 | 0.9927 | 0.7085 |
| Left Sulcus, Orbital Medial (Olfactory) | 0.7950 | 0.7919 | 0.9933 | 0.6403 |
| Right Sulcus, Orbital Medial (Olfactory) | 0.8564 | 0.9397 | 0.9117 | 0.6598 |
| Left Sulcus, Orbital H-Shaped | 0.9486 | 0.9480 | 0.9830 | 0.7186 |
| Right Sulcus, Orbital H-Shaped | 0.9378 | 0.9472 | 0.9750 | 0.6963 |
| Left Sulcus, Parieto-occipital | 0.9658 | 0.9936 | 0.9960 | 0.8513 |
| Right Sulcus, Parieto-occipital | 0.7925 | 0.9730 | 0.9558 | 0.6862 |
| Left Sulcus, Pericallosal | 0.9066 | 0.9910 | 0.9760 | 0.8252 |
| Right Sulcus,Pericallosal | 0.9152 | 0.9107 | 0.9246 | 0.7775 |
| Left Sulcus, Postcentral | 0.9854 | 0.9827 | 0.9815 | 0.8252 |
| Right Sulcus, Postcentral | 0.9790 | 0.9917 | 0.9891 | 0.8236 |
| Left Sulcus, Precentral Inferior Part | 0.9804 | 0.9844 | 0.9822 | 0.7405 |
| Right Sulcus, Precentral Inferior Part | 0.9798 | 0.9900 | 0.9925 | 0.8849 |
| Left Sulcus, Precentral Superior Part | 0.9773 | 0.9915 | 0.9932 | 0.8062 |
| Right Sulcus, Precentral Superior Part | 0.9705 | 0.9862 | 0.9910 | 0.7284 |
| Left Sulcus, Suborbital | 0.9561 | 0.9747 | 0.9873 | 0.7319 |
| Right Sulcus, Suborbital | 0.9330 | 0.9497 | 0.9698 | 0.7262 |
| Left Sulcus, Subparietal | 0.9443 | 0.9957 | 0.9965 | 0.8921 |
| Right Sulcus, Subparietal | 0.9655 | 0.9762 | 0.9619 | 0.7762 |
| Left Sulcus, Temporal Inferior | 0.9704 | 0.9266 | 0.9777 | 0.6978 |
| Right Sulcus, Temporal Inferior | 0.9567 | 0.9863 | 0.9902 | 0.7411 |
| Left Sulcus, Temporal Superior | 0.9785 | 0.8722 | 0.9607 | 0.7321 |
| Right Sulcus, Temporal Superior | 0.9808 | 0.9912 | 0.9872 | 0.8934 |
| Left Sulcus, Temporal Transverse | 0.9725 | 0.8717 | 0.8072 | 0.6730 |
| Right Sulcus, Temporal Transverse | 0.9746 | 0.9860 | 0.9766 | 0.7704 |
|  |  |  |  |  |

**Table S2.** [Table caption].

| Brain Region | cortical thickness | | fractal dimension | | sulcus depth | | gyrification index | |
| --- | --- | --- | --- | --- | --- | --- | --- | --- |
|  | t-statistic | p value | t-statistic | p value | t-statistic | p value | t-statistic | p value |
| Left Gyrus and Sulcus, Frontal Margin | -8.7625 | 0.0000 | -0.4705 | 0.6394 | 0.8248 | 0.4122 | -0.6507 | 0.5173 |
| Right Gyrus and Sulcus, Frontal Margin | -6.8201 | 0.0000 | -1.6341 | 0.1065 | 0.5935 | 0.5547 | 2.1601 | 0.0340 |
| Left Gyrus and Sulcus, Occipital Inferior | -0.2486 | 0.8044 | -0.7001 | 0.4861 | 0.4168 | 0.6780 | -0.7720 | 0.4426 |
| Right Gyrus and Sulcus, Occipital Inferior | -4.5381 | 0.0000 | -1.3240 | 0.1896 | 2.4566 | 0.0164 | -2.9569 | 0.0042 |
| Left Gyrus and Sulcus, Paracentral | -5.3479 | 0.0000 | 2.7634 | 0.0072 | 0.8996 | 0.3713 | -1.0497 | 0.2973 |
| Right Gyrus and Sulcus, Paracentral | -2.8959 | 0.0050 | -0.4118 | 0.6817 | 0.0285 | 0.9773 | 0.2356 | 0.8144 |
| Left Gyrus and Sulcus, Subcentral | -2.7678 | 0.0071 | -2.3325 | 0.0224 | 9.9260 | 0.0000 | -0.8823 | 0.3805 |
| Right Gyrus and Sulcus, Subcentral | -4.4401 | 0.0000 | 2.7717 | 0.0071 | 1.5243 | 0.1318 | -2.2245 | 0.0292 |
| Left Gyrus and Sulcus, Transversal Frontal Pole | -3.8895 | 0.0002 | 3.3212 | 0.0014 | 1.3086 | 0.1948 | -2.3364 | 0.0222 |
| Right Gyrus and Sulcus, Transversal Frontal Pole | -4.3801 | 0.0000 | 2.4586 | 0.0163 | 0.4416 | 0.6601 | 1.7018 | 0.0931 |
| Left Gyrus and Sulcus, Anterior Cingulate | -10.2776 | 0.0000 | 4.6185 | 0.0000 | 1.9412 | 0.0561 | 0.2455 | 0.8068 |
| Right Gyrus and Sulcus, Anterior Cingulate | -8.4400 | 0.0000 | 0.8283 | 0.4102 | 9.2643 | 0.0000 | -0.7490 | 0.4562 |
| Left Gyrus and Sulcus, Mid Anterior Cingulate | -3.8747 | 0.0002 | 3.1104 | 0.0027 | 1.4409 | 0.1539 | -2.5872 | 0.0117 |
| Right Gyrus and Sulcus, Mid Anterior Cingulate | -5.3728 | 0.0000 | -3.3880 | 0.0011 | 9.2858 | 0.0000 | -0.1672 | 0.8676 |
| Left Gyrus and Sulcus, Mid Posterior Cingulate | -7.0401 | 0.0000 | 0.3239 | 0.7469 | 1.6172 | 0.1102 | -0.3217 | 0.7486 |
| Right Gyrus and Sulcus, Mid Posterior Cingulate | -5.7417 | 0.0000 | 0.8026 | 0.4248 | 12.2718 | 0.0000 | -2.5278 | 0.0136 |
| Left Cingulate, Posterior Dorsal | -2.2349 | 0.0285 | 1.6107 | 0.1116 | 1.3321 | 0.1870 | -0.7127 | 0.4783 |
| Right Cingulate, Posterior Dorsal | -0.0510 | 0.9595 | -0.5011 | 0.6178 | 10.4848 | 0.0000 | 0.6307 | 0.5302 |
| Left Cingulate, Posterior Ventral | -7.4294 | 0.0000 | -2.8193 | 0.0062 | 2.2410 | 0.0281 | -1.0645 | 0.2906 |
| Right Cingulate, Posterior Ventral | -4.2883 | 0.0001 | -1.6116 | 0.1114 | 2.4864 | 0.0152 | -1.7726 | 0.0805 |
| Left Gyrus, Cuneus | 1.0845 | 0.2817 | 1.2698 | 0.2082 | 1.3707 | 0.1747 | -0.7686 | 0.4446 |
| Right Gyrus, Cuneus | 3.8114 | 0.0003 | -3.4242 | 0.0010 | 5.1240 | 0.0000 | -1.0095 | 0.3161 |
| Left Gyrus, Inferior Frontal Opercular | 2.6993 | 0.0086 | 0.7411 | 0.4610 | 9.1438 | 0.0000 | 0.8376 | 0.4050 |
| Right Gyrus, Inferior Frontal Opercular | 3.4749 | 0.0009 | 2.9879 | 0.0038 | 1.7012 | 0.0932 | -1.7213 | 0.0894 |
| Left Gyrus, Inferior Frontal Orbital | -4.2818 | 0.0001 | -0.7244 | 0.4711 | 3.3674 | 0.0012 | 0.8701 | 0.3871 |
| Right Gyrus, Inferior Frontal Orbital | -3.1204 | 0.0026 | -1.4460 | 0.1525 | 0.7078 | 0.4813 | -0.0389 | 0.9691 |
| Left Gyrus, Inferior Frontal Triangular | 0.9524 | 0.3440 | 2.6865 | 0.0089 | 5.6810 | 0.0000 | 1.8096 | 0.0745 |
| Right Gyrus, Inferior Frontal Triangular | -0.8438 | 0.4015 | -1.0263 | 0.3081 | 1.2684 | 0.2087 | -2.5192 | 0.0140 |
| Left Gyrus, Middle Frontal | 3.1124 | 0.0026 | 0.4542 | 0.6510 | 2.0183 | 0.0472 | -2.1652 | 0.0336 |
| Right Gyrus, Middle Frontal | 2.5145 | 0.0141 | 5.8177 | 0.0000 | -1.2817 | 0.2040 | -3.1670 | 0.0022 |
| Left Gyrus, Superior Frontal | 1.9974 | 0.0495 | 8.2709 | 0.0000 | 2.0545 | 0.0435 | -3.4349 | 0.0010 |
| Right Gyrus, Superior Frontal | 3.0194 | 0.0035 | 7.1166 | 0.0000 | 6.8077 | 0.0000 | -1.5610 | 0.1228 |
| Left Insula, Large and Central Insular Sulcus | -3.6228 | 0.0005 | 1.6889 | 0.0955 | 2.0526 | 0.0437 | -1.0575 | 0.2938 |
| Right Insula, Large and Central Insular Sulcus | -8.2578 | 0.0000 | -3.5943 | 0.0006 | 3.9159 | 0.0002 | 1.3961 | 0.1669 |
| Left Insula, Short Gyrus | -1.8962 | 0.0619 | 0.1651 | 0.8694 | 2.2616 | 0.0267 | -0.2047 | 0.8384 |
| Right Insula, Short Gyrus | -3.1025 | 0.0027 | -2.9081 | 0.0048 | -1.0846 | 0.2816 | 1.2623 | 0.2109 |
| Left Gyrus, Occipital Middle | -6.6704 | 0.0000 | 1.3961 | 0.1669 | 2.5656 | 0.0124 | -1.2892 | 0.2014 |
| Right Gyrus, Occipital Middle | -8.6221 | 0.0000 | 4.6708 | 0.0000 | 1.0805 | 0.2835 | -1.8276 | 0.0717 |
| Left Gyrus, Occipital Superior | -4.0624 | 0.0001 | 0.2395 | 0.8114 | -0.9676 | 0.3364 | -0.5249 | 0.6012 |
| Right Gyrus, Occipital Superior | -3.0477 | 0.0032 | 0.9681 | 0.3362 | -0.1098 | 0.9129 | -1.1378 | 0.2589 |
| Left Occipital-Temporal, Lateral Fusiform | 4.1940 | 0.0001 | 1.0856 | 0.2812 | 0.3193 | 0.7504 | -1.4989 | 0.1382 |
| Right Occipital-Temporal, Lateral Fusiform | 2.7822 | 0.0069 | -2.5270 | 0.0137 | -1.2872 | 0.2021 | -1.6246 | 0.1086 |
| Left Occipital-Temporal, Medial Lingual | 9.7502 | 0.0000 | 0.6706 | 0.5046 | 4.4942 | 0.0000 | -0.0578 | 0.9541 |
| Right Occipital-Temporal, Medial Lingual | 9.7156 | 0.0000 | 0.9227 | 0.3592 | 4.3869 | 0.0000 | -2.8458 | 0.0057 |
| Left Occipital-Temporal, Medial Parahippocampal | -11.3401 | 0.0000 | -0.2154 | 0.8301 | 3.9031 | 0.0002 | 0.6115 | 0.5428 |
| Right Occipital-Temporal, Medial Parahippocampal | -9.6653 | 0.0000 | -2.9150 | 0.0047 | -0.1264 | 0.8998 | 0.6134 | 0.5415 |
| Left Gyrus, Orbital | -6.3981 | 0.0000 | -2.7587 | 0.0073 | 3.7428 | 0.0004 | -1.6298 | 0.1074 |
| Right Gyrus, Orbital | -7.3311 | 0.0000 | -3.5328 | 0.0007 | 4.3611 | 0.0000 | -0.3899 | 0.6978 |
| Left Parietal Inferior, Angular | -1.1857 | 0.2396 | -0.0168 | 0.9866 | 2.9491 | 0.0043 | -1.3765 | 0.1729 |
| Right Parietal Inferior, Angular | -1.3777 | 0.1725 | -0.4320 | 0.6670 | -1.0101 | 0.3158 | -0.1560 | 0.8764 |
| Left Parietal Inferior, Supramarginal | -1.8318 | 0.0711 | 0.7506 | 0.4553 | 11.3648 | 0.0000 | 0.7001 | 0.4861 |
| Right Parietal Inferior, Supramarginal | -5.2103 | 0.0000 | 3.5150 | 0.0008 | 1.4643 | 0.1474 | -0.5590 | 0.5779 |
| Left Parietal Superior | 4.0551 | 0.0001 | 3.2372 | 0.0018 | 0.4340 | 0.6656 | 0.5769 | 0.5658 |
| Right Parietal Superior | 3.3325 | 0.0014 | 4.6976 | 0.0000 | -1.2869 | 0.2022 | 0.9441 | 0.3482 |
| Left Gyrus, Postcentral | -3.9144 | 0.0002 | 5.8112 | 0.0000 | 5.1779 | 0.0000 | -2.3334 | 0.0224 |
| Right Gyrus, Postcentral | -3.2086 | 0.0020 | 4.5633 | 0.0000 | -2.2003 | 0.0310 | -0.4941 | 0.6227 |
| Left Gyrus, Precentral | 0.2341 | 0.8156 | 2.6371 | 0.0102 | 6.0702 | 0.0000 | -3.3174 | 0.0014 |
| Right Gyrus, Precentral | -0.2877 | 0.7744 | 2.9190 | 0.0047 | 3.6300 | 0.0005 | -2.7068 | 0.0085 |
| Left Gyrus, Precuneus | 2.0088 | 0.0483 | 3.4391 | 0.0010 | 0.7234 | 0.4717 | -0.4542 | 0.6510 |
| Right Gyrus, Precuneus | 1.9884 | 0.0505 | -1.2052 | 0.2320 | 3.3672 | 0.0012 | 0.4039 | 0.6875 |
| Left Gyrus, Rectus | -6.5889 | 0.0000 | -0.0701 | 0.9443 | 0.2468 | 0.8058 | 1.5514 | 0.1251 |
| Right Gyrus, Rectus | -5.3447 | 0.0000 | -0.3878 | 0.6993 | 3.8704 | 0.0002 | 0.7208 | 0.4733 |
| Left Gyrus, Subcallosal | -2.2756 | 0.0258 | -2.4546 | 0.0165 | 2.6141 | 0.0109 | 2.1704 | 0.0332 |
| Right Gyrus, Subcallosal | -0.8621 | 0.3914 | -3.3990 | 0.0011 | 3.1984 | 0.0020 | -2.1104 | 0.0382 |
| Left Temporal Superior, G_T Transversal | 10.3980 | 0.0000 | 3.1703 | 0.0022 | -8.0233 | 0.0000 | 5.8990 | 0.0000 |
| Right Temporal Superior, G_T Transversal | 5.6740 | 0.0000 | -1.8306 | 0.0712 | 0.3378 | 0.7365 | -1.5140 | 0.1343 |
| Left Temporal Superior, Lateral | -3.6930 | 0.0004 | 2.0246 | 0.0466 | 3.1294 | 0.0025 | -0.0243 | 0.9807 |
| Right Temporal Superior, Lateral | -2.5847 | 0.0117 | -3.5119 | 0.0008 | 2.1706 | 0.0332 | -1.4905 | 0.1404 |
| Left Temporal Superior, Planar Polar | 0.5404 | 0.5906 | 0.7621 | 0.4485 | 0.3618 | 0.7185 | 1.3457 | 0.1825 |
| Right Temporal Superior, Planar Polar | 1.0458 | 0.2991 | 1.9028 | 0.0610 | 2.5007 | 0.0146 | -1.3268 | 0.1887 |
| Left Temporal Superior, Planar Temporal | -5.2215 | 0.0000 | 2.8734 | 0.0053 | 7.4322 | 0.0000 | 5.0777 | 0.0000 |
| Right Temporal Superior, Planar Temporal | -2.8589 | 0.0055 | 3.9910 | 0.0002 | -1.1922 | 0.2371 | -2.3610 | 0.0209 |
| Left Gyrus, Temporal Inferior | -11.8799 | 0.0000 | -3.8074 | 0.0003 | -1.0417 | 0.3010 | -3.8973 | 0.0002 |
| Right Gyrus, Temporal Inferior | -14.7728 | 0.0000 | 1.1862 | 0.2394 | 2.7917 | 0.0067 | 1.4787 | 0.1435 |
| Left Gyrus, Temporal Middle | -8.4758 | 0.0000 | -0.6377 | 0.5256 | 1.1288 | 0.2627 | -2.9103 | 0.0048 |
| Right Gyrus, Temporal Middle | -7.4595 | 0.0000 | 5.6303 | 0.0000 | 3.8949 | 0.0002 | -1.1925 | 0.2369 |
| Left Lateral Fissure, Anterior Horizontal | -3.4586 | 0.0009 | 0.4105 | 0.6826 | 2.4703 | 0.0158 | 2.2383 | 0.0283 |
| Right Lateral Fissure, Anterior Horizontal | 0.1269 | 0.8994 | -0.9609 | 0.3398 | 0.0874 | 0.9306 | -1.4294 | 0.1572 |
| Left Lateral Fissure, Anterior Vertical | -3.0478 | 0.0032 | 1.9858 | 0.0508 | 5.0958 | 0.0000 | -2.3100 | 0.0237 |
| Right Lateral Fissure, Anterior Vertical | -1.9841 | 0.0510 | 1.0850 | 0.2815 | 0.5743 | 0.5675 | 0.7506 | 0.4553 |
| Left Lateral Fissure, Posterior | -5.4716 | 0.0000 | -4.8721 | 0.0000 | -6.1067 | 0.0000 | -0.1227 | 0.9027 |
| Right Lateral Fissure, Posterior | -5.9110 | 0.0000 | -1.8513 | 0.0682 | 2.3575 | 0.0211 | 0.9371 | 0.3518 |
| Left Occipital Pole | -6.6136 | 0.0000 | 4.1264 | 0.0001 | 3.3701 | 0.0012 | -3.9258 | 0.0002 |
| Right Occipital Pole | -5.9958 | 0.0000 | 0.8096 | 0.4208 | 2.4197 | 0.0180 | -0.9993 | 0.3209 |
| Left Temporal Pole | -8.4339 | 0.0000 | 1.1342 | 0.2604 | 0.8925 | 0.3751 | 1.8627 | 0.0665 |
| Right Temporal Pole | -7.8161 | 0.0000 | 4.5928 | 0.0000 | 4.5681 | 0.0000 | 0.4432 | 0.6589 |
| Left Sulcus, Calcarine | 0.3572 | 0.7220 | 0.1065 | 0.9155 | 2.4320 | 0.0175 | -2.5673 | 0.0123 |
| Right Sulcus, Calcarine | 4.2870 | 0.0001 | 0.9053 | 0.3683 | 1.0109 | 0.3154 | -2.3986 | 0.0190 |
| Left Sulcus, Central | -2.7245 | 0.0081 | 7.5837 | 0.0000 | 8.4466 | 0.0000 | -4.7483 | 0.0000 |
| Right Sulcus, Central | -2.1686 | 0.0334 | 9.8083 | 0.0000 | 4.5264 | 0.0000 | -3.9130 | 0.0002 |
| Left Sulcus, Cingulate Marginalis | -12.0852 | 0.0000 | 2.2767 | 0.0257 | 0.3993 | 0.6909 | -0.5892 | 0.5576 |
| Right Sulcus, Cingulate Marginalis | -10.7597 | 0.0000 | 0.1514 | 0.8801 | 5.0590 | 0.0000 | 0.5320 | 0.5964 |
| Left Sulcus, Circular Insula Anterior | -3.1658 | 0.0023 | -3.8956 | 0.0002 | 3.2849 | 0.0016 | 0.6490 | 0.5184 |
| Right Sulcus, Circular Insula Anterior | -4.6217 | 0.0000 | 1.9766 | 0.0519 | 1.4198 | 0.1599 | 0.1979 | 0.8437 |
| Left Sulcus, Circular Insula Inferior | -2.9582 | 0.0042 | -1.6026 | 0.1133 | 3.4908 | 0.0008 | -1.7581 | 0.0829 |
| Right Sulcus, Circular Insula Inferior | -4.6824 | 0.0000 | 5.7629 | 0.0000 | 4.0587 | 0.0001 | -0.6903 | 0.4922 |
| Left Sulcus, Circular Insula Superior | -15.7905 | 0.0000 | -2.7580 | 0.0073 | 6.6505 | 0.0000 | 0.2965 | 0.7677 |
| Right Sulcus, Circular Insula Superior | -15.1988 | 0.0000 | 0.7243 | 0.4712 | 2.5387 | 0.0133 | 2.3416 | 0.0219 |
| Left Sulcus, Collateral Transversal Anterior | -0.9760 | 0.3323 | -3.7033 | 0.0004 | -1.4894 | 0.1407 | -0.7900 | 0.4321 |
| Right Sulcus, Collateral Transversal Anterior | -6.1546 | 0.0000 | -3.5604 | 0.0007 | -0.7937 | 0.4299 | -0.0887 | 0.9296 |
| Left Sulcus, Collateral Transversal Posterior | -0.2302 | 0.8186 | -2.5450 | 0.0130 | -0.3783 | 0.7063 | -1.7998 | 0.0760 |
| Right Sulcus, Collateral Transversal Posterior | 4.2660 | 0.0001 | -1.8915 | 0.0625 | -2.6087 | 0.0110 | -2.2898 | 0.0249 |
| Left Sulcus, Inferior Frontal | -7.4114 | 0.0000 | 5.1836 | 0.0000 | 0.2281 | 0.8202 | -0.2935 | 0.7699 |
| Right Sulcus, Inferior Frontal | -9.3036 | 0.0000 | 6.2855 | 0.0000 | 2.8185 | 0.0062 | -4.6383 | 0.0000 |
| Left Sulcus, Middle Frontal | -5.0009 | 0.0000 | 3.8013 | 0.0003 | -1.8060 | 0.0750 | -2.8456 | 0.0057 |
| Right Sulcus, Middle Frontal | -5.6704 | 0.0000 | 0.6818 | 0.4975 | 0.6039 | 0.5478 | -1.7696 | 0.0810 |
| Left Sulcus, Superior Frontal | -2.3545 | 0.0212 | 4.0211 | 0.0001 | 1.1225 | 0.2653 | -1.9349 | 0.0569 |
| Right Sulcus, Superior Frontal | -0.4812 | 0.6318 | 0.9202 | 0.3605 | 2.9290 | 0.0045 | -1.2589 | 0.2121 |
| Left Sulcus, Intermittent Primary Jensen | -3.9476 | 0.0002 | -0.7048 | 0.4832 | 4.4843 | 0.0000 | 0.6584 | 0.5123 |
| Right Sulcus, Intermittent Primary Jensen | -5.4479 | 0.0000 | 2.6614 | 0.0096 | 1.0838 | 0.2820 | 0.6742 | 0.5023 |
| Left Sulcus, Intraparietal and Parietal Transversal | -14.5718 | 0.0000 | 2.2165 | 0.0298 | -0.6316 | 0.5296 | 2.1819 | 0.0323 |
| Right Sulcus, Intraparietal and Parietal Transversal | -10.7587 | 0.0000 | 1.7925 | 0.0772 | 1.9864 | 0.0507 | 1.6196 | 0.1096 |
| Left Sulcus, Occipital Middle and Lunatus | -9.0090 | 0.0000 | 1.6273 | 0.1080 | 1.5741 | 0.1198 | -1.6930 | 0.0947 |
| Right Sulcus, Occipital Middle and Lunatus | -8.6793 | 0.0000 | 2.1968 | 0.0312 | 4.3074 | 0.0001 | -4.1589 | 0.0001 |
| Left Sulcus, Occipital Superior and Transversal | -11.8145 | 0.0000 | 2.0050 | 0.0487 | 3.3967 | 0.0011 | -1.6285 | 0.1077 |
| Right Sulcus, Occipital Superior and Transversal | -16.4496 | 0.0000 | 0.4201 | 0.6757 | 3.4662 | 0.0009 | -2.9951 | 0.0037 |
| Left Sulcus, Occipital Anterior | -3.6113 | 0.0006 | 4.4655 | 0.0000 | 3.2527 | 0.0017 | 0.8924 | 0.3751 |
| Right Sulcus, Occipital Anterior | -4.5004 | 0.0000 | 1.3885 | 0.1692 | 2.4951 | 0.0149 | -2.1613 | 0.0340 |
| Left Sulcus, Occipital-Temporal Lateral | -4.1931 | 0.0001 | -1.6352 | 0.1063 | -2.1682 | 0.0334 | -2.7961 | 0.0066 |
| Right Sulcus, Occipital-Temporal Lateral | -3.9029 | 0.0002 | 2.1026 | 0.0389 | 3.4082 | 0.0011 | 0.0000 | 1.0000 |
| Left Sulcus, Occipital-Temporal Medial and Lingual | -10.0692 | 0.0000 | 3.2442 | 0.0018 | 2.6491 | 0.0099 | 0.2396 | 0.8113 |
| Right Sulcus, Occipital-Temporal Medial and Lingual | -9.2720 | 0.0000 | -2.9042 | 0.0049 | -3.4747 | 0.0009 | -3.0877 | 0.0029 |
| Left Sulcus, Orbital Lateral | -3.7172 | 0.0004 | -0.0444 | 0.9647 | 1.0331 | 0.3049 | 1.3293 | 0.1879 |
| Right Sulcus, Orbital Lateral | -4.2163 | 0.0001 | 4.9335 | 0.0000 | 0.3140 | 0.7544 | -1.6184 | 0.1099 |
| Left Sulcus, Orbital Medial (Olfactory) | -3.5449 | 0.0007 | -1.5391 | 0.1281 | 2.3484 | 0.0216 | 1.0446 | 0.2996 |
| Right Sulcus, Orbital Medial (Olfactory) | -5.2830 | 0.0000 | -1.8983 | 0.0616 | 3.1861 | 0.0021 | 1.3428 | 0.1835 |
| Left Sulcus, Orbital H-Shaped | -8.1772 | 0.0000 | 2.4469 | 0.0168 | 4.3878 | 0.0000 | 0.2010 | 0.8413 |
| Right Sulcus, Orbital H-Shaped | -7.5892 | 0.0000 | 2.9454 | 0.0043 | 4.7168 | 0.0000 | -1.0857 | 0.2812 |
| Left Sulcus, Parieto-occipital | -5.5425 | 0.0000 | 4.4482 | 0.0000 | 3.5161 | 0.0008 | 0.1196 | 0.9051 |
| Right Sulcus, Parieto-occipital | 0.1981 | 0.8435 | -0.5811 | 0.5630 | 3.1677 | 0.0022 | 1.3676 | 0.1756 |
| Left Sulcus, Pericallosal | -14.9068 | 0.0000 | 0.9392 | 0.3507 | 0.6260 | 0.5333 | 0.3292 | 0.7430 |
| Right Sulcus,Pericallosal | -15.4151 | 0.0000 | -5.4424 | 0.0000 | 4.9931 | 0.0000 | 1.0551 | 0.2949 |
| Left Sulcus, Postcentral | -8.9530 | 0.0000 | 0.7035 | 0.4840 | 2.4155 | 0.0182 | 0.6628 | 0.5095 |
| Right Sulcus, Postcentral | -8.7531 | 0.0000 | 3.1307 | 0.0025 | 0.9908 | 0.3251 | -0.5306 | 0.5973 |
| Left Sulcus, Precentral Inferior Part | -6.6447 | 0.0000 | 4.8647 | 0.0000 | 7.2264 | 0.0000 | 0.1262 | 0.8999 |
| Right Sulcus, Precentral Inferior Part | -5.9325 | 0.0000 | 4.6990 | 0.0000 | 3.2847 | 0.0016 | -1.9824 | 0.0512 |
| Left Sulcus, Precentral Superior Part | -1.3423 | 0.1836 | 1.5170 | 0.1336 | 3.1085 | 0.0027 | -0.0022 | 0.9982 |
| Right Sulcus, Precentral Superior Part | -2.0700 | 0.0420 | 2.3950 | 0.0192 | 5.5419 | 0.0000 | -2.0517 | 0.0438 |
| Left Sulcus, Suborbital | -3.2460 | 0.0018 | -0.8385 | 0.4045 | 2.0409 | 0.0449 | -0.5406 | 0.5905 |
| Right Sulcus, Suborbital | -1.8192 | 0.0730 | -1.2612 | 0.2112 | 4.7544 | 0.0000 | -0.7858 | 0.4345 |
| Left Sulcus, Subparietal | -4.3539 | 0.0000 | 3.7900 | 0.0003 | 2.0905 | 0.0401 | 0.1300 | 0.8969 |
| Right Sulcus, Subparietal | -5.1063 | 0.0000 | 0.6645 | 0.5084 | 6.8728 | 0.0000 | 1.4035 | 0.1647 |
| Left Sulcus, Temporal Inferior | -7.9317 | 0.0000 | -2.4022 | 0.0188 | 0.4212 | 0.6748 | -3.1851 | 0.0021 |
| Right Sulcus, Temporal Inferior | -8.4622 | 0.0000 | 3.3358 | 0.0013 | 4.5891 | 0.0000 | 0.0232 | 0.9816 |
| Left Sulcus, Temporal Superior | -14.7017 | 0.0000 | -2.0204 | 0.0470 | 1.6113 | 0.1114 | -0.5899 | 0.5571 |
| Right Sulcus, Temporal Superior | -19.0206 | 0.0000 | 1.7561 | 0.0833 | 3.4619 | 0.0009 | -1.7305 | 0.0878 |
| Left Sulcus, Temporal Transverse | 4.1495 | 0.0001 | 4.3094 | 0.0001 | -3.1993 | 0.0020 | 7.2404 | 0.0000 |
| Right Sulcus, Temporal Transverse | 7.4441 | 0.0000 | -1.2099 | 0.2302 | -0.1254 | 0.9005 | -2.0385 | 0.0451 |
